# Diagnosis of Ovarian Tumors with Special Reference to the Operation of Ovariotomy

**Published:** 1872-12

**Authors:** 


*Diagnosis of Ovarian Tumors, with special reference to the operation of Ovariotomy and Occasional Pathological and Therapeutical Considerations.* By WASHINGTON L. ALLEE, M. D. Philadelphia: J. B. Lippincott & Co. Buffalo: Martin Taylor.

This book is mainly a clinical record of the experience of the author, and contains numerous cases in detail, a careful study of which is instructive. Nearly every form of ovarian tumor is included in the report and the best methods of diagnosis fully described.

The work will be found a valuable guide to those who are not familiar with such disease, as obscure subjects are illustrated and all explained by detail of cases. Perhaps the method of the author in writing his book to illustrate his subjects with cuts and illustrative cases might be imitated with advantage.

